# Structural analysis of a bacterial UDP-sugar 2-epimerase reveals the active site architecture before and after catalysis

**DOI:** 10.1016/j.jbc.2023.105200

**Published:** 2023-09-03

**Authors:** James B. Thoden, James O. McKnight, Charles W. Kroft, Joshua D.T. Jast, Hazel M. Holden

**Affiliations:** Department of Biochemistry, University of Wisconsin, Madison, Wisconsin, USA

**Keywords:** bacteria, carbohydrate, crystallography, 2,3-diacetamido-2,3-dideoxy-d-mannuronic acid, enzyme structure, lipopolysaccharide, O-antigen

## Abstract

The sugar, 2,3-diacetamido-2,3-dideoxy-d-mannuronic acid, was first identified ∼40 years ago in the O-antigen of *Pseudomonas aeruginosa* O:3,a,d. Since then, it has been observed on the O-antigens of various pathogenic Gram-negative bacteria including *Bordetella pertussis*, *Escherichia albertii,* and *Pseudomonas mediterranea.* Previous studies have established that five enzymes are required for its biosynthesis beginning with uridine dinucleotide (UDP)-*N*-acetyl-d-glucosamine (UDP-GlcNAc). The final step in the pathway is catalyzed by a 2-epimerase, which utilizes UDP-2,3-diacetamido-2,3-dideoxy-d-glucuronic acid as its substrate. Curious as to whether this biochemical pathway is found in extreme thermophiles, we examined the published genome sequence for *Thermus thermophilus* HB27 and identified five ORFs that could possibly encode for the required enzymes. The focus of this investigation is on the ORF WP_011172736, which we demonstrate encodes for a 2-epimerase. For this investigation, ten high resolution X-ray crystallographic structures were determined to resolutions of 2.3 Å or higher. The models have revealed the manner in which the 2-epimerase anchors its UDP-sugar substrate as well as its UDP-sugar product into the active site. In addition, this study reveals for the first time the manner in which any sugar 2-epimerase can simultaneously bind UDP-sugars in both the active site and the allosteric binding region. We have also demonstrated that the *T. thermophilus* enzyme is allosterically regulated by UDP-GlcNAc. Whereas the sugar 2-epimerases that function on UDP-GlcNAc have been the focus of past biochemical and structural analyses, this is the first detailed investigation of a 2-epimerase that specifically utilizes UDP-2,3-diacetamido-2,3-dideoxy-d-glucuronic acid as its substrate.

2,3-Diacetamido-2,3-dideoxy-d-mannuronic acid, shown in Figure 1 and hereafter referred to as ManNAc3NAcA, is a rare bacterial sugar first identified over 40 years ago in the B-band O-antigen of *Pseudomonas aeruginosa* O:3,a,d (1). Since then, it has been observed, for example, in additional *P. aeruginosa* species, in the lipopolysaccharide of *Bordetella pertussis*, the causative agent of whopping cough, in the O-antigen of *Escherichia albertii*, an emerging foodborne pathogen, and in the O-antigen of *Pseudomonas mediterranea* strain C5P1rad1, the bacterium responsible for tomato pith necrosis (2, 3, 4, 5, 6).

**Figure 1:**
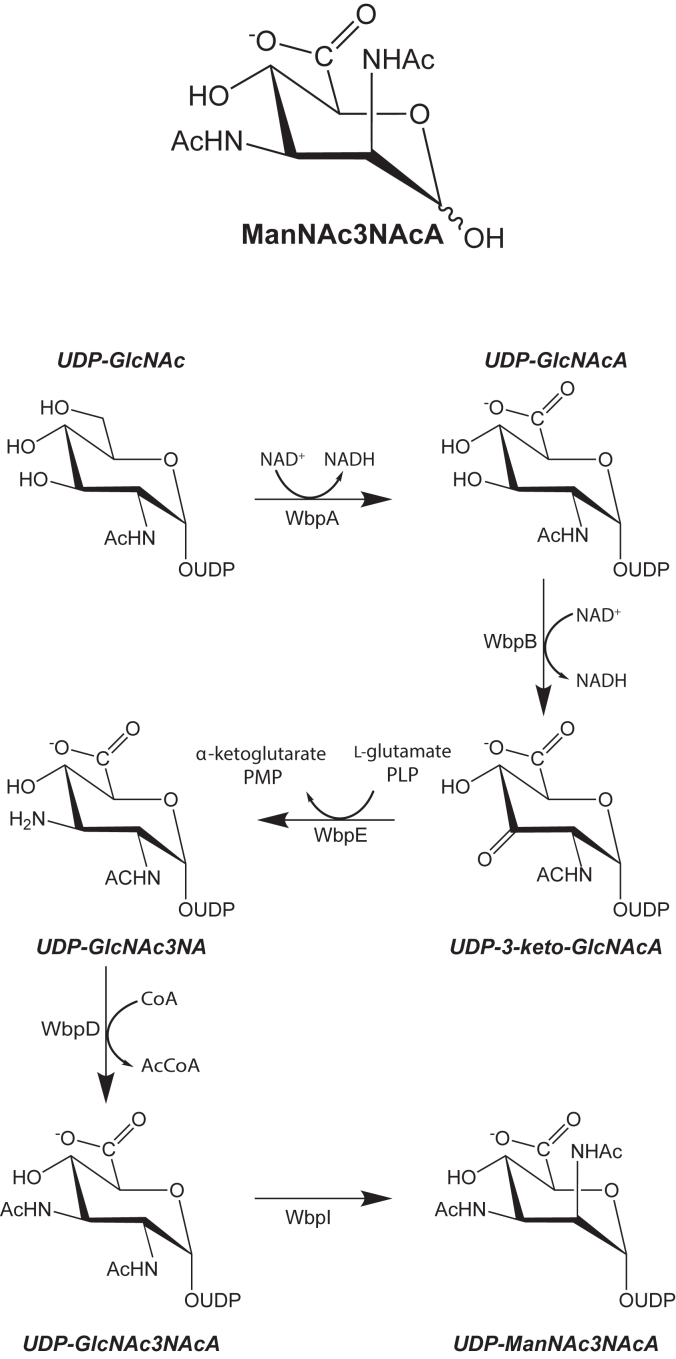
**Pathway for the production of UDP-ManNAc3NAcA**.

On the basis of the elegant studies reported by the Lam laboratory, five enzymes have been identified for the biosynthesis of ManNAc3NAcA in *P. aeruginosa* PAO1 as outlined in Figure 1 (3, 7). The first step involves the oxidation of the C-6′ carbon of uridine dinucleotide-*N*-acetyl-d-glucosamine (UDP-GlcNAc) by an NAD^+^-dependent dehydrogenase (WbpA), thereby leading to the formation of UDP-*N*-acetyl-d-glucosaminuronic acid (UDP-GlcNAcA). In the following step the C-3′ carbon is oxidized by another NAD^+^-dependent enzyme referred to as WbpB, yielding UDP-3-keto-*N*-acetyl-d-glucosaminuronic acid (UDP-3-keto-GlcNAcA). Subsequently, amination of the C-3′ keto functionality *via* the action of WbpE, a pyridoxal-5′-phosphate dependent enzyme, results in the formation of UDP-2-acetamido-3-amino-2,3-dideoxy-d-glucuronic acid (UDP-GlcNAc3NA). In the penultimate step of the biosynthetic pathway, UDP-GlcNAc3NA is converted to UDP-2,3-diacetamido-2,3-dideoxy-d-glucuronic acid (UDP-GlcNAc3NAcA) by WbpD, an *N*-acetyltransferase whose three-dimensional structure was determined in this laboratory (8). The final enzyme in the pathway, WbpI, catalyzes an epimerization reaction about the C-2′ carbon of the hexose, resulting in the formation of UDP-ManNAc3NAcA. Interestingly, in some organisms such as *P. aeruginosa* O:3a,d, WbpB functions *via* a ping-pong mechanism employing α-ketoglutarate as the NADH oxidant. In other organisms, however, such as *B. pertussis* and *Chromobacterium violaceum*, the equivalent enzymes function *via* sequential mechanisms (9, 10, 11).

Given our past interest in the enzymes involved in the biosynthesis of ManNAc3NAcA, we were curious as to whether this sugar is found in the extreme thermophile *Thermus thermophilus* HB27. Accordingly, we examined its genome for ORFs that might encode for the required enzymes (12). Strikingly, members of the genus *Thermus* can grow at temperatures ranging from 50° to 75° (13, 14), and unlike typical Gram-negative bacteria, their outer membranes possess unusual glycolipids rather than lipopolysaccharides (15).

We identified potential genes in *T. thermophilus* HB27. Listed in Table 1 are the gene designations, the enzymes that are hypothetically encoded by them, and the amino acid sequence similarities and identities to those proteins first identified in the Lam laboratory. From research in this laboratory, we have already demonstrated that the gene WP_011172738 encodes for a 3-dehydrogenase (10).

**Table 1:** Amino acid sequence identities and similarities between the *P*.*aeruginosa* and the *T*. *thermophilus* enzymes involved in UDP-ManNAc3NAcA biosynthesis

Enzyme name in *P. aeruginosa* PAO1	Gene name in *T. thermophilus* HB27	Hypothetical enzymatic activity in *T. thermophilus*	Amino acid sequence identity to *P. aeruginosa* PAO1 (%)	Amino acid sequence similarity to *P. aeruginosa* PAO1 (%)
WbpA	WP_011172739	6-dehydrogenase	49	68
WbpB	WP_011172738	3-dehydrogenase	49	67
WbpE	WP_148187554	3-aminotransferase	49	65
WbpD	WP_011172737	*N*-acetyltransferase	60	75
WbpI	WP_011172736	2-epimerase	55	70

The focus of this investigation is on the protein encoded by WP_011172736, which hereafter will be referred to as the *T. thermophilus* 2-epimerase. Previous X-ray crystallographic studies have demonstrated that the 2-epimerases which function on UDP-GlcNAc rather than UDP-GlcNAc3NAcA are homodimers. These epimerases demonstrate significant structural homology with glycogen phosphorylase and T4 phage β-glucosyltransferase (16, 17, 18). Their reaction mechanisms, as indicated in Figure 2, involve an *anti*-elimination of UDP thereby generating a 2-acetamidoglucal intermediate followed by the *syn*-addition of UDP to produce UDP-ManNAc (19, 20, 21). In keeping with their reaction mechanisms, these enzymes are referred to in the literature as “non-hydrolyzing” 2-epimerases. Intriguingly, many of these bacterial epimerases are allosterically regulated with their substrate, UDP-GlcNAc, functioning as an activator (21, 22, 23).

**Figure 2:**
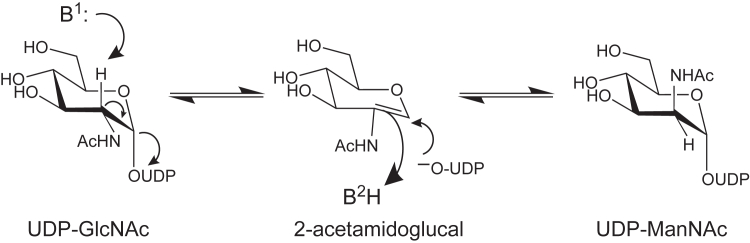
**Proposed catalytic mechanism for nonhydrolyzing 2-epimerases**.

Herein, we describe a detailed structural analysis of the *T. thermophilus* 2-epimerase which functions on UDP-GlcNAc3NAcA rather than UDP-GlcNAc. Importantly, for the first time, the manner in which a 2-epimerase can accommodate UDP-sugars in both the active site and the allosteric binding pocket has been revealed.

## Results

### Structure of the *T. thermophilus* 2-epimerase

**Figure 3:**
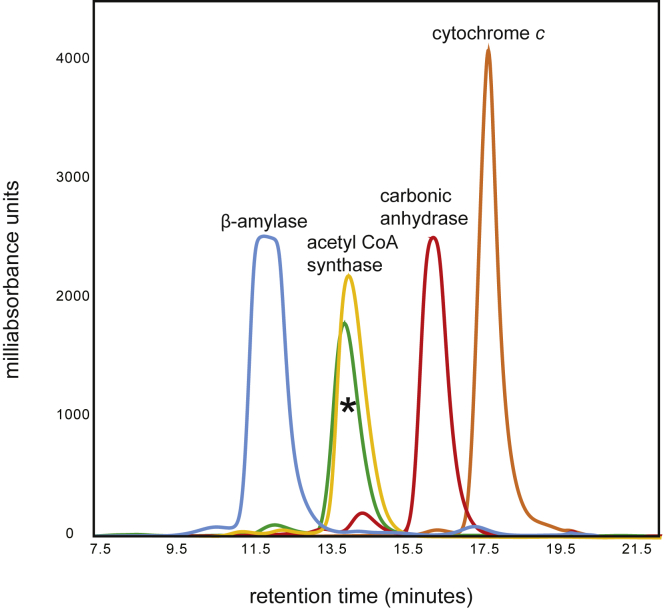
**Chromatogram from sizing analysis.** Gel-filtration chromatography was utilized to confirm the quaternary structure of the enzyme. Shown is a graph of milliabsorbance units *versus* minutes. The molecular weights of the standards are β-amylase (200,000), acetyl CoA synthase (73,000), carbonic anhydrase (29,000), and cytochrome *c* (12,000). The *asterisk* marks the curve corresponding to the 2-epimerase (82,000).

The first structure determined in this investigation was that of the apoenzyme crystallized at pH 9.0 (Table 2, structure 1). The crystals of the protein belonged to the *P*2_1_ space group with two subunits in the asymmetric unit. The model was refined at 2.3 Å resolution with an overall *R*-factor of 20.1%. Relevant refinement statistics for this structure and all those presented below are provided in Table 2. The two subunits superimpose with an RMSD of 0.6 Å. On the basis of gel-filtration chromatography, the *T. thermophilus* 2-epimerase assumes a dimeric quaternary structure (Fig. 3).

**Table 2:** Crystallization, X-ray data collection statistics and model refinement statistics

PDB code	Structure 1	Structure 2	Structure 3	Structure 4	Structure 5
	8SXV	8SXY	8SY0	8SY9	8SYA
Crystallization data					
Protein (histidine tag)	WT enzyme (none)	WT enzyme (C-terminal)	WT enzyme (C-terminal)	D98N variant (C-terminal)	D98N variant (C-terminal)
pH (100 mmM buffer)	9.0 (CHES)	5.0 (Homo-Pipes)	9.0 (CHES)	7.0 (MOPS)	9.0 (CHES)
Precipitant	8–12% poly(ethylene glycol) 8000 + 1 M NMe_4_Cl	22–27% pentaerythritol propoxylate (5/4 P O/OH)	18–22% poly(ethylene glycol) 3350 + 200 mM LiCl	22–27% pentaerythritol propoxylate (5/4 PO/OH)	18–22% poly(ethylene glycol) 8000
Ligands used in crystallization	none	UDP-GlcNAc3NAcA	UDP-GlcNAc3NAcA	UDP-GlcNAc3NAcA	UDP-GlcNAc3NAcA
Ligands in structure	none	UDP-ManNAc3NAcA	UDP-ManNAc3NAcA	UDP-GlcNAc3NAcA, UDP-GlcNAc, UDP	UDP-GlcNAc3NAcA, UDP
Unit Cell					
a, b, c (Å)	61.7, 88.9, 87.6	56.5, 128.8, 58.7	56.3, 128.6, 58.9	56.5, 128.4, 58.5	56.1, 130.0, 58.6
α, β, γ (deg)	β = 98.8	β = 111.1	β = 111.2	β = 112.6	β = 112.6
Space group	*P*2_1_	*P*2_1_	*P*2_1_	*P*2_1_	*P*2_1_
Data collection statistics					
Resolution limits (Å)	50–2.3 (2.4–2.3)^a^	50–1.8 (1.9–1.8)^a^	50–2.1 (2.2–2.1)^a^	50–2.2 (2.3–2.2)^a^	50–2.3 (2.4–2.3)^a^
Number of independent reflections	41,084 (4732)	71,545 (10,677)	45,153 (5715)	38,323 (4569)	34,302 (4050)
Completeness (%)	98.7 (96.2)	99.0 (98.7)	99.1 (96.9)	98.1 (94.4)	99.6 (98.8)
Redundancy	8.6 (4.7)	7.4 (4.4)	7.9 (3.5)	7.4 (3.3)	8.5 (3.8)
Avg I/avg σ(I)	13.0 (2.6)	17.5 (3.5)	14.7 (2.7)	12.8 (2.4)	9.7 (2.2)
*R*_sym_ (%)^b^	8.1 (45.1)	5.6 (30.4)	8.2 (36.3)	9.5 (46.5)	9.6 (44.7)
Refinement statistics					
*R*-factor (overall)%/no. reflections^c^	20.1/41,084	18.2/71,545	18.3/45,153	19.5/38,323	20.4/34,302
*R*-factor (working)%/no. reflections	19.8/39,026	17.9/68,030	18.0/42,938	19.2/36,412	20.2/32,597
*R*-factor (free)%/no. reflections	25.9/2058	21.8/3515	23.1/2215	25.9/1911	24.3/1705
Number of protein atoms	5700	5717	5700	5679	5676
Number of heteroatoms	203	576	378	301	262
Average B values					
Protein atoms (Å^2^)	39.9	22.1	27.5	33.4	30.7
Ligand (Å^2^)	n/a	12.0	16.5	34.4	26.4
Solvent (Å^2^)	28.8	29.2	27.1	25.2	20.3
Weighted RMS deviations from ideality					
Bond lengths (Å)	0.008	0.008	0.009	0.007	0.007
Bond angles (º)	1.51	1.56	1.62	1.52	1.49
Planar groups (Å)	0.007	0.008	0.008	0.007	0.006
Ramachandran regions (%)^d^					
Most favored	96.1	98.0	96.5	96.4	95.5
Additionally allowed	3.3	1.7	3.2	2.9	4.2
Generously allowed	0.6	0.3	0.3	0.7	0.3


PDB code	Structure 6	Structure 7	Structure 8	Structure 9	Structure 10
	8SYB	8SYD	8SYE	8SYH	8SXW
Crystallization data					
Protein (histidine tag)	D98N variant (C-terminal)	D98N variant (N-terminal)	D98N variant (C-terminal)	D98N variant (C-terminal)	D98N variant (none)
pH (buffer)	9.0 (CHES)	6.0 (MES)	6.0 (MES)	8.0 (HEPPS)	6.0 (MES)
Precipitant	18–22% poly(ethylene glycol) 8000 + 200 mM KCl	22–27% pentaerythritol ethoxylate (three-fourths EO/OH)	22–27% pentaerythritol propoxylate (5/4 PO/OH)	22–27% pentaerythritol propoxylate (5/4 PO/OH)	18–22% pentaerythritol propoxylate (5/4 PO/OH)
Ligands used in crystallization	UDP-GlcNAc3NAcA, UDP-GlcNAc	UDP-GlcNAc3NAcA, UDP-GlcNAc	UDP-GlcNAc3NAcA, UDP	UDP-GlcNAc3NAcA, UDP	none
Ligands in structure	UDP-GlcNAc3NAcA, UDP-GlcNAc	UDP-GlcNAc3NAcA, UDP-GlcNAc	UDP-GlcNAc3NAcA, UDP	UDP-GlcNAc3NAcA, UDP	none
Unit Cell					
a, b, c (Å)	55.8, 128.2, 58.5	56.3, 128.4, 58.1	56.7, 128.1, 58.4	56.8, 128.2, 58.5	64.3, 85.3, 86.5
α, β, γ (deg)	β = 113.6	β = 114.1	β = 112.6	β = 112.5	α = 108.2, β = 110.1, γ = 97.1
Space group	*P*2_1_	*P*2_1_	*P*2_1_	*P*2_1_	*P*1
Data collection statistics					
Resolution limits (Å)	50–2.2 (2.3–2.2)^a^	50–2.2 (2.3–2.2)^a^	50–1.9 (2.0–1.9)^a^	50–2.0 (2.1–2.0)^a^	50–1.8 (1.9–1.8)^a^
Number of independent reflections	38,034 (4689)	38,022 (4649)	58,879 (7452)	51,464 (6828)	139,158 (19,632)
Completeness (%)	99.4 (98.4)	99.5 (97.9)	97.5 (86.8)	98.9 (96.6)	96.1 (89.3)
Redundancy	7.2 (3.7)	7.9 (3.7)	6.4 (2.3)	6.4 (3.3)	4.6 (2.4)
Avg I/avg σ(I)	10.7 (2.3)	12.8 (3.2)	11.1 (2.4)	12.1 (2.0)	16.5 (2.7)
*R*_sym_ (%)^b^	8.4 (38.6)	8.5 (37.9)	9.7 (44.2)	9.0 (43.5)	5.5 (35.6)
Refinement statistics					
*R*-factor (overall)%/no. reflections^c^	19.4/38,034	17.5/38,022	20.0/58,879	18.6/51,464	18.1/139,158
*R*-factor (working)%/no. reflections	19.1/36,209	17.2/36,200	19.8/56,098	18.3/48,754	17.8/132,025
*R*-factor (free)%/no. reflections	25.2/1825	22.9/1822	23.2/2781	23.4/2710	22.4/7133
Number of protein atoms	5684	5759	5714	5697	11,445
Number of heteroatoms	393	440	439	435	1114
Average B values					
Protein atoms (Å^2^)	26.4	16.9	25.9	30.2	27.0
Ligand (Å^2^)	16.2	8.6	21.3	26.4	n/a
Solvent (Å^2^)	21.1	17.3	27.1	31.2	32.0
Weighted RMS deviations from ideality					
Bond lengths (Å)	0.007	0.008	0.008	0.009	0.007
Bond angles (º)	1.45	1.60	1.53	1.60	1.42
Planar groups (Å)	0.006	0.007	0.008	0.008	0.007
Ramachandran regions (%)^d^					
Most favored	97.4	97.4	97.4	97.2	98.8
Additionally allowed	2.5	2.6	2.5	2.5	1.5
Generously allowed	0.1	0.0	0.1	0.3	0.3

a Statistics for the highest resolution bin.

b R_sym_ = (∑|I - I^–^ |/∑ I) × 100.

c R-factor = (Σ|*F*_o_ − *F*_c_|/Σ|*F*_o_|) × 100 where *F*_o_ is the observed structure-factor amplitude and *F*_c_ is the calculated structure-factor amplitude.

d Distribution of Ramachandran angles according to PROCHECK (35).

**Figure 4:**
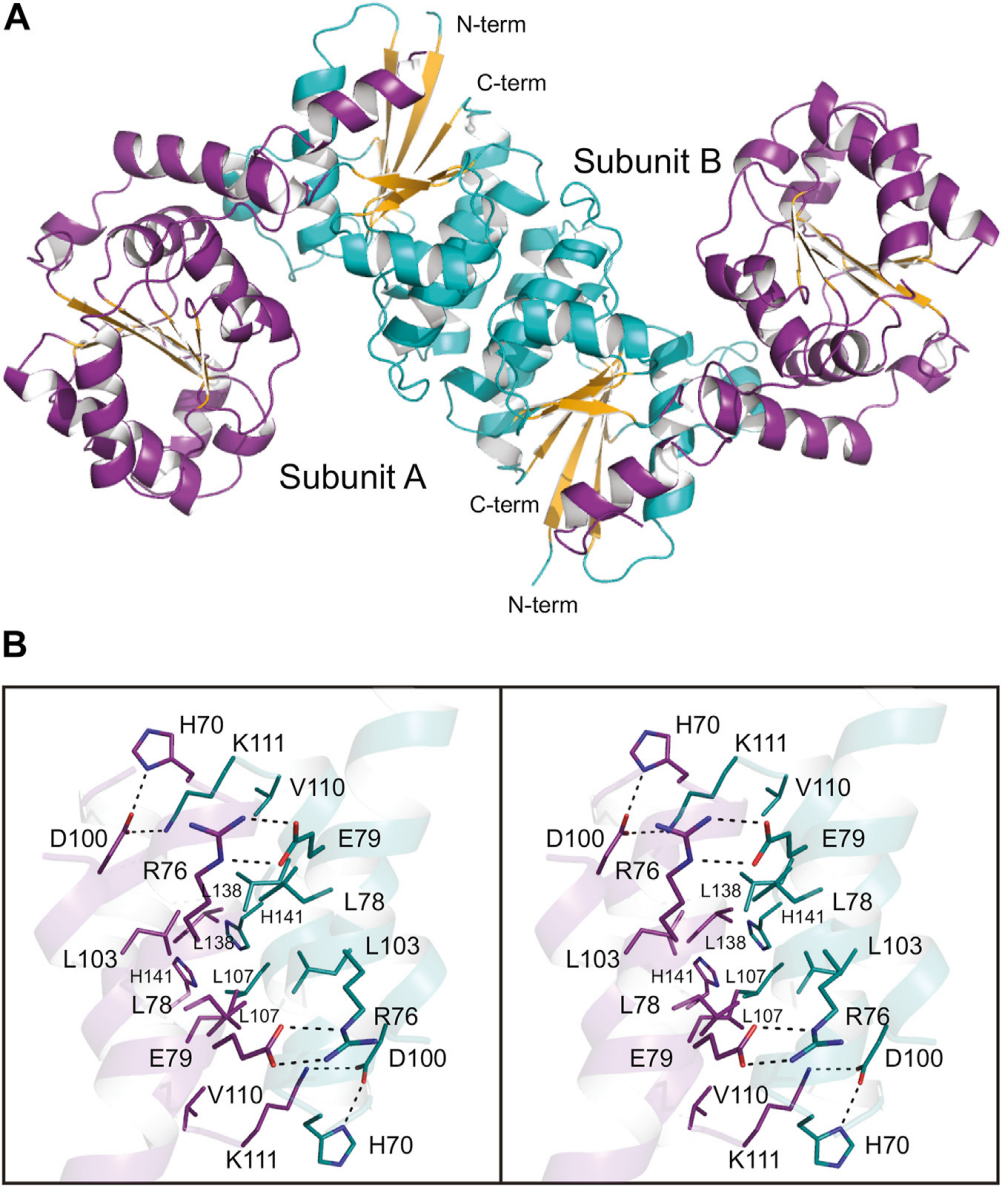
**Structure of the *T. thermophilus* 2-epimerase (structure 1, PDB****8SXV****).** Shown in (*A*) is a ribbon representation of the apoenzyme dimer as observed in the asymmetric unit. Each individual subunit adopts a bilobal architecture with the N-terminal domain constructed around a seven-stranded parallel β-sheet and the C-terminal domain consisting of a six-stranded parallel β-sheet. The N- and C-terminal domains are highlighted in *teal* and *dark violet*, respectively. The subunit:subunit interface is primarily hydrophobic in nature as can be seen in (*B*). This figure and Figure 5, Figure 6, Figure 7, Figure 8, Figure 9, Figure 11, and 12B were prepared with PyMol (http://www.pymol.org/pymol). PDB, Protein Data Bank.

A ribbon representation of the dimer is provided in Figure 4*A*. Each subunit adopts a bilobal architecture with domain 1 defined by Met 1 to Val 170 and domain 2 formed by Gly 171 to Gly 346. The architecture of domain 1 is dominated by a seven-stranded parallel β-sheet flanked on either side by a total of six α-helices. Domain 2 contains a six-stranded parallel β-sheet surrounded by nine α-helices. The final α-helix, formed by Ala 350 to Lys 360, extends from domain 2 back to domain 1. As a result, the α-carbons for the N- and C-termini are separated by only ∼13 Å.

The total buried surface area for the dimer is ∼2850 Å^2^. The subunit:subunit interface is primarily formed by three α-helices defined by His 70 to Glu 88, Thr 99 to Lys 111, and Pro 131 to Ala 142 in subunit A running antiparallel to the same α-helices in subunit B. As shown in Figure 4*B*, the interface is fairly hydrophobic in nature with Leu 78, Leu 103, Leu 107, Val 110, and Leu 138 projecting inward. In addition, there are hydrogen bonding interactions between Asp 100 in one subunit and Lys 111 in the other (and vice versa). One end of the interface is capped off by a salt bridge between Arg 76 in one subunit and Glu 79 in the other (and vice versa) whereas at the other end the side chains of His 141 from each subunit participate in a parallel stacking interaction with one another.

### Structure of the *T. thermophilus* 2-epimerase/product complexes

**Figure 5:**
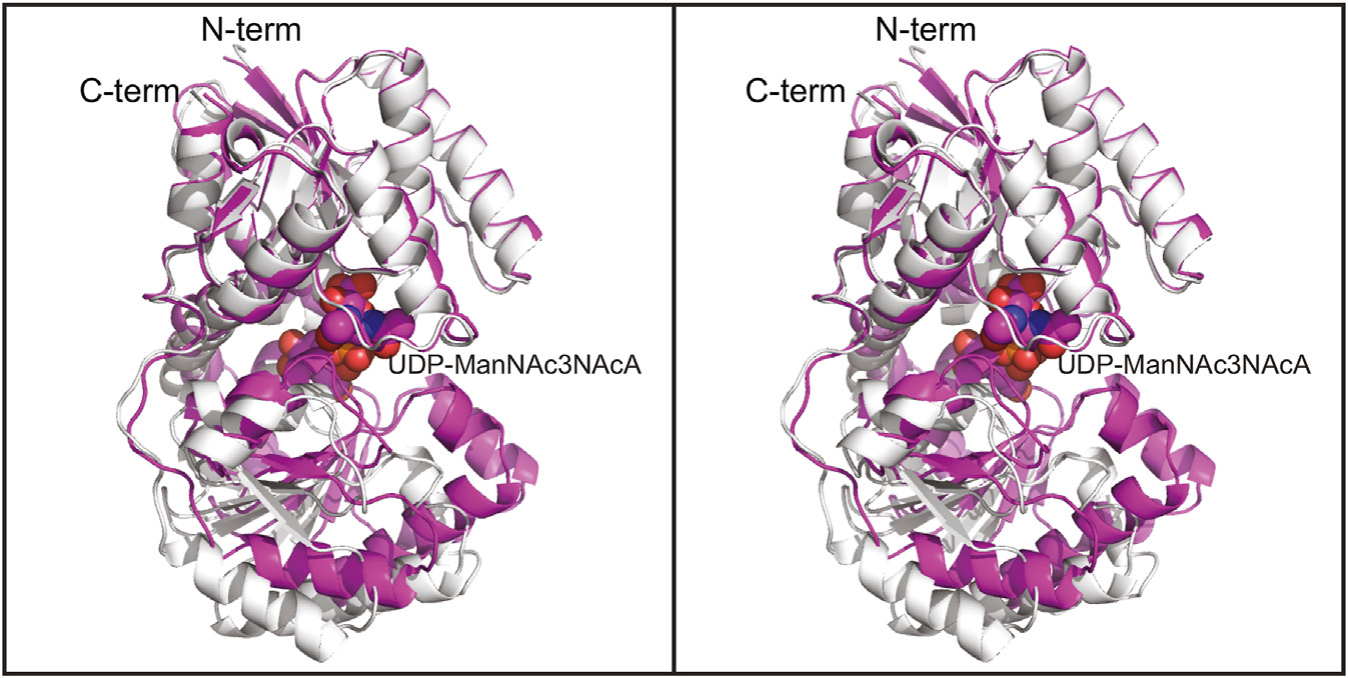
**Domain movement upon UDP-sugar binding to the *T***. ***thermophilus* 2-epimerase.** When a UDP-ligand is trapped in the active site, the N- and C-terminal domains move toward one another as shown here. The *ribbon* representations for the apoenzyme and the protein/UDP-ManNAc3NAcA complex are colored in *light blue* and *purple violet*, respectively. The UDP-ManNAc3NAcA is shown in *sphere* representation. To highlight the domain movement, the N-terminal domains for both structures were aligned *via* least-squares in Coot (31, 32) and utilizing the α-carbons for Met 1 to Gly 171.

The next structure solved in this investigation was that of the WT enzyme crystallized in the presence of its substrate, UDP-GlcNAc3NAcA at pH 5.0 (Table 2, structures 2 and 3). The asymmetric unit contained a dimer. The model was refined at 1.8 Å resolution with an overall *R*-factor of 18.2%. The α-carbons for the two subunits superimpose with an RMSD of 0.8 Å. As observed for other 2-epimerases, there is a significant closure of the gap between the N- and C-terminal domains upon UDP-sugar binding as highlighted in Figure 5. Indeed, some α-carbons move by ∼11 Å.

**Figure 6:**
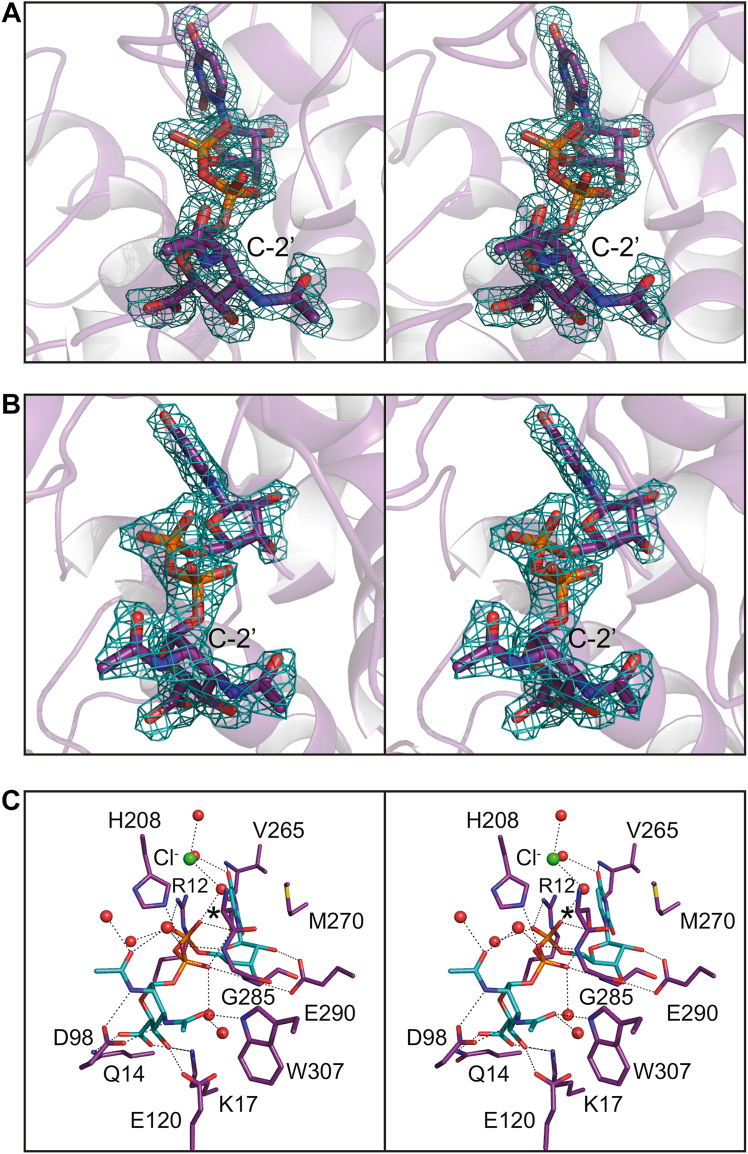
**Structure of the *T***. ***thermophilus* 2-epimerase with bound UDP-ManNAc3NAcA.** Shown in stereo in (*A*) is the observed electron density corresponding to the UDP-sugar ligand in subunit A with crystals grown at pH 5.0 (structure 2, PDB 8SXY).The map was calculated with (*F*_*o*_–*F*_*c*_) coefficients and contoured at 3σ. The ligand was not included in the X-ray coordinate file used to calculate the omit map, and thus there is no model bias. It is clear that UDP-ManNAc3NAcA was trapped in the active site. Shown in stereo in (*B*) is the observed electron density corresponding to the UDP-sugar ligand in subunit A with crystals grown at pH 9.0 (structure 3, PDB 8SY0). The map was calculated with (*F*_*o*_–*F*_*c*_) coefficients and contoured at 3σ. Again, UDP-ManNAc3NAcA was trapped in the active site region. A close-up stereo view of the active site in subunit A at pH 5.0 is presented in (*C*). The UDP-product and amino acid side chains are highlighted in *teal* and *purple* bonds, respectively. *Dashed lines* indicate potential hydrogen bonds within 3.2 Å. Ordered water molecules are depicted as *red spheres*. The chloride ion is colored in *green*. The position of Ser 284 is indicated by the *asterisk*. PDB, Protein Data Bank.

As can be seen in Figure 6*A*, whereas UDP-GlcNAc3NAcA was utilized in the crystallization trials, the product, namely UDP-ManNAc3NAcA, was observed in each subunit. Curious as to whether the product rather than substrate was trapped in the active site due to the pH of the crystallization conditions, we next determined the structure of the WT enzyme using crystals grown at pH 9.0 (2.1 Å resolution, overall *R*-factor of 18.3%). Again, the product was observed in the active sites of the dimer (Fig. 6*B*). The α-carbons for subunits A determined at pH 5.0 and pH 9.0 superimpose with an RMSD of 0.2 Å and their active site geometries are nearly identical within experimental error. The only exception is that the chloride observed in the structure solved at pH 5.0 is replaced by a water molecule at pH 9.0.

A close-up stereo view of the active site in subunit A with bound UDP-product (pH 5.0) is presented in Figure 6*C*. The uracil ring is positioned into the active site by the backbone amide and carbonyl oxygen of Val 265 and a water molecule. Glu 290 lies within hydrogen bonding distance to the ribosyl oxygens. The pyrophosphoryl moiety is surrounded by the side chains of Arg 12, His 208, and Ser 284. There are also four water molecules and the backbone amide groups of Gly 285 and Gly 286 that lie within 3.2 Å of the phosphoryl groups. The pyranosyl moiety is anchored into the active site by the side chains of Gln 14, Lys 17, Asp 98, Glu 120, and Trp 307. In addition, there are three water molecules surrounding the ManNAc3NAcA group. In both subunits A and B, there is a chloride ion located within ∼3.2 Å of the side chain of Arg 12.

### Structure of the *T. thermophilus* 2-epimerase/substrate complexes

From previous investigations on the 2-epimerases, we expected that Asp 98 would play a key role in catalysis (19, 20, 21) (Table 2, structures 4, 5, 6, 7, 8, and 9). In an attempt to trap the UDP-sugar substrate in the active site, we subsequently constructed the D98N variant and crystallized it at pH 7.0 in the presence of UDP-GlcNAc3NAcA (structure 4). Again, the asymmetric unit contained a dimer (α-carbons of the two subunits superimpose with an RMSD of 0.4 Å). The model was refined to an overall *R*-factor of 19.5% at 2.2 Å resolution.

**Figure 7:**
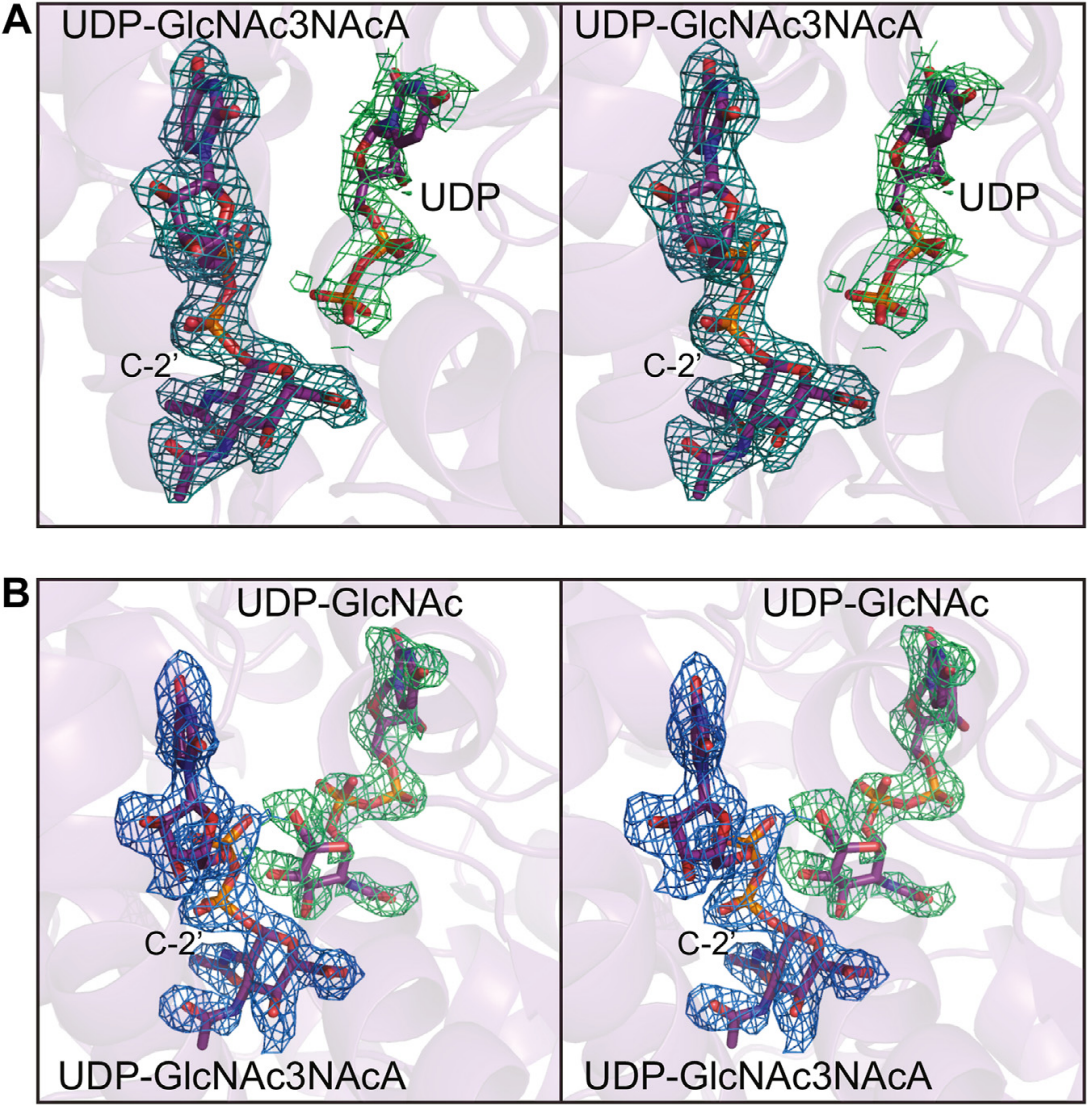
**Structure of the *T***. ***thermophilus* 2-epimerase D98N variant (structure 4, PDB 2-epimerase D98N variant (structure 4, PDB****8SY9****).** Shown in (*A*) is the electron density corresponding to UDP-GlcNAc3NAcA and UDP in subunit A. In subunit B, there was electron density for both UDP-GlcNAc3NAcA and UDP-GlcNAc as can be seen in (*B*). The omit maps were calculated as described in the legend to Figure 6. PDB, Protein Data Bank. UDP-GlcNAc, UDP-*N*-acetyl-d-glucosamine.

Unambiguous electron density corresponding to the UDP-substrate was observed in each subunit. Surprisingly, however, in subunits A and B, there were also electron densities corresponding to UDP and UDP-GlcNAc, respectively, albeit at lower occupancies as can be seen in Figure 7, *A* and *B*. Importantly, these ligands were not included in the crystallization trials. The UDP in subunit A and the UDP-GlcNAc in subunit B align closely with the exception of their β-phosphoryl groups. In subunit A, the β-phosphoryl group does not lie within 3.2 Å of any protein atoms, whereas in subunit B, it forms an electrostatic interaction with Arg 242. The UDP-GlcNAc3NAcA moieties assume identical conformations in both subunits within experimental error.

To determine whether the pH of the crystallization experiments was a contributing factor toward the binding of additional ligands, the next structure (Table 2, Structure 5) was determined at pH 9.0. Again, the crystals obtained were grown in the presence UDP-GlcNAc3NAcA but in the absence of additional ligands. The model was refined to an overall *R*-factor of 20.4% at 2.3 Å resolution. Both subunits contained UDP-GlcNAc3NAcA in their active sites. In subunit A, the auxiliary binding pocket was empty whereas in subunit B, electron density corresponding to UDP was observed. The α-carbons for the dimer models determined at either pH 7.0 or pH 9.0 superimpose with an RMSD of 0.2 Å.

**Figure 8:**
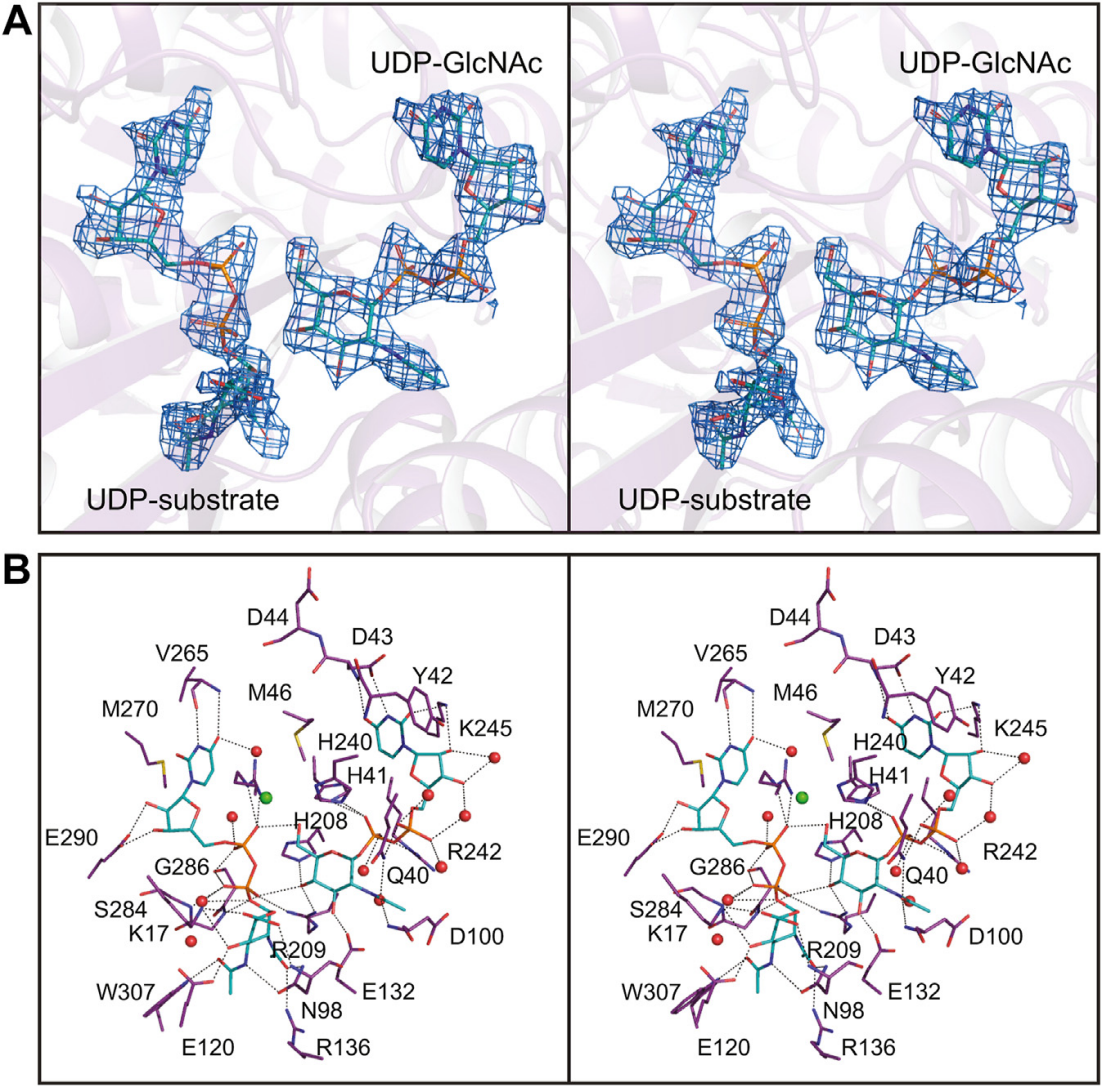
**Structure of the *T***. ***thermophilus* 2-epimerase D98N variant with both UDP-substrate and UDP-GlcNAc bound (structure 6, PDB****8SYB****).** Shown in (*A*) is the electron density corresponding to the ligands bound to subunit A in structure 6. The omit map was calculated as described in the legend to Figure 6. A close-up stereo view of the active site is provided in (*B*). The ligands and the protein side chains are highlighted in *teal* and *violet*, respectively. Ordered water molecules are depicted as *red spheres*. Interactions between the ligands and the protein within 3.2 Å are indicated by the *dashed lines*. PDB, Protein Data Bank; UDP-GlcNAc, UDP-*N*-acetyl-d-glucosamine.

Given the unexpected presence of UDP-GlcNAc in structure 4, we next conducted crystallization trials that included both UDP-GlcNAc3NAcA and UDP-GlcNAc. Crystals were obtained at pH 9.0 (structure 6) and pH 6.0 (structure 7). Both models at pH 9.0 and 6.0 were determined to 2.2 Å resolution and refined to overall *R*-factors of 19.4% and 17.5%, respectively. Regardless of pH, each subunit of the dimer contained both UDP-GlcNAc3NAcA in the active site and UDP-GlcNAc in the auxiliary binding pocket. Representative electron densities for the ligands in the model solved at pH 9.0 are shown in Figure 8*A*.

A close-up view of the active site for subunit A (pH 9.0) is provided in Figure 8*B*. As can be seen, extensive hydrogen bonding occurs between the UDP-sugar ligands and the protein. There are two direct interactions between the UDP-sugar ligands: one between C-4′ of UDP-GlcNAc and a β-phosphoryl oxygen of UDP-GlcNAc3NAcA (3.2 Å) and another one between C-6′ of UDP-GlcNAc and an α-phosphoryl oxygen of UDP-GlcNAc3NAcA (3.0 Å). The side chain of Arg 209 serves as a link between the two ligands. Those side chains responsible for anchoring UDP-GlcNAc into the allosteric pocket include Gln 40, His 41, Asp 43, Glu 132, His 208, Arg 209, His 240, Arg 242, and Lys 245. The conservation of these residues in the 2-epimerases will be discussed in more detail below.

**Figure 9:**
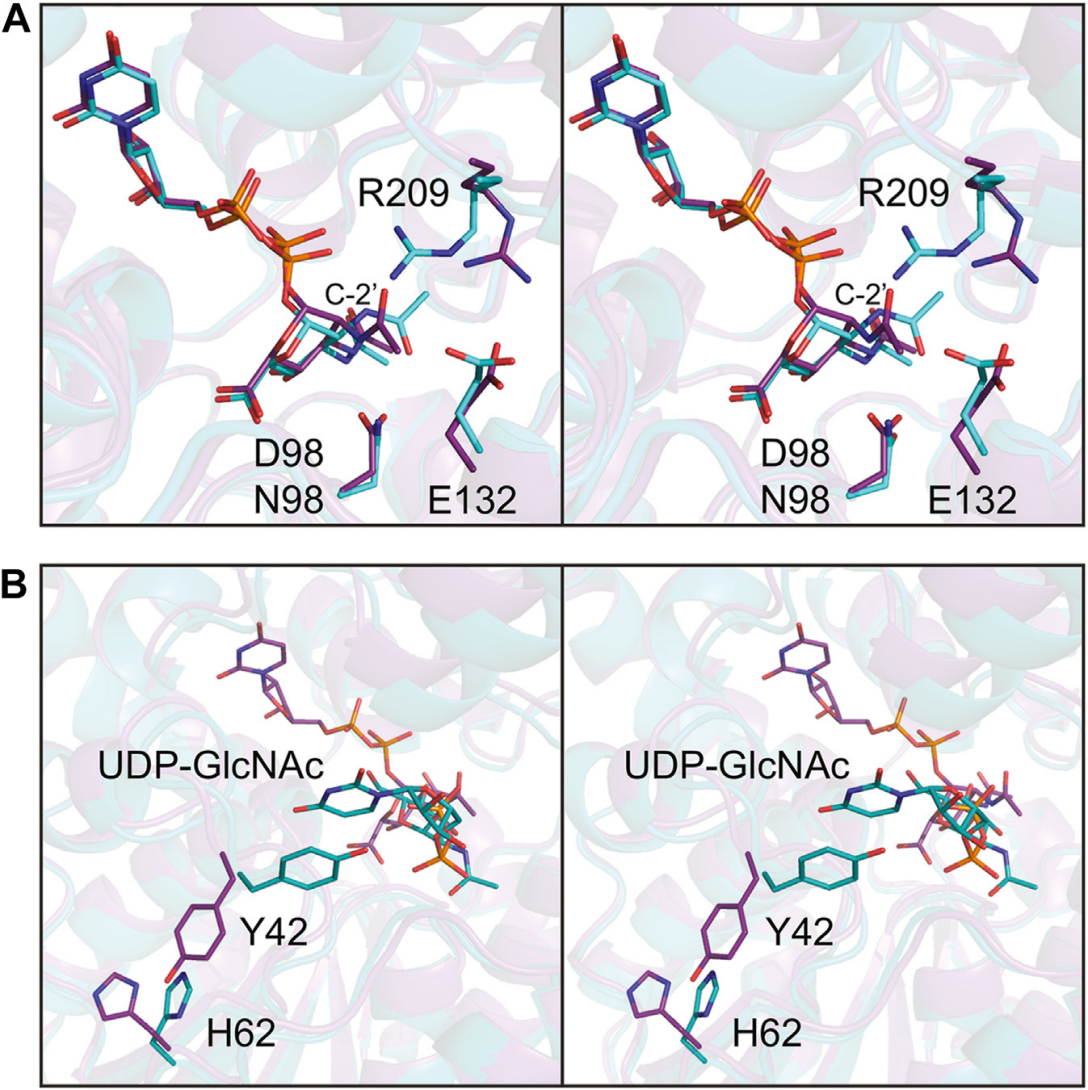
**Comparison of the *T***. ***thermophilus* 2-epimerase with bound UDP-ManNAc3NAcA or UDP-GlcNAc3NAcA.** As shown in (*A*), there are only two significant side chain movements when the two different UDP-sugars are bound. The models with UDP-ManNAc3NAcA and UDP-GlcNAc3NAcA are highlighted in *teal* and *violet*, respectively. When UDP-GlcNAc binds in the allosteric pocket, the side chain of Tyr 42 swings into the pocket to form a parallel stacking interaction with the uracil ring as can be seen in (*B*). Structure 3 is highlighted with *violet* bonds whereas structure 6 is displayed in *teal* bonds. UDP-GlcNAc, UDP-*N*-acetyl-d-glucosamine.

Shown in Figure 9*A* is a close-up stereo view of the 2-epimerase active site with either UDP-GlcNAc3NAcA (structure 3) or UDP-ManNAc3NAcA (structure 6) bound. Both structures were determined at pH 9.0. The α-carbons for the two dimers correspond with an RMSD of 1.0 Å. The only significant differences are those observed with the side chains of Glu 132 and Arg 209 that move to accommodate the different configuration of the *N*-acetyl group at the C-2′ carbon. In structure 3, the N^η1^ and N^η2^ atoms of Arg 209 lie within ∼3 Å to the O^ε1^ of Glu 132. This interaction is disrupted in structure 6, whereby the side chain of Arg 209 swings toward a phosphoryl oxygen of the UDP-ligand.

As noted previously, structure 6 contains UDP-GlcNAc in the allosteric binding pocket whereas structure 3 does not. The only significant difference between the two models involves Tyr 42 and His 62. As displayed in Figure 9*B*, the side chain of Tyr 42 swings into the allosteric pocket to aid in anchoring the UDP moiety to the protein *via* parallel aromatic stacking interactions. In the absence of UDP-GlcNAc, the side chain of Tyr 42 projects toward the surface which in turn displaces the side chain of His 62. Indeed, the electron density for Tyr 42 in structure 3 is weak.

For structures 8 and 9 (Table 2), crystals were grown in the presence of UDP-GlcNAc3NAcA and UDP at either pH 6.0 or pH 8.0. Regardless of the pH, UDP-GlcNAc3NAcA was bound in the active site and UDP in the auxiliary binding pocket.

### Structure of the *T. thermophilus* D98N variant in the absence of ligands

Although UDP-GlcNAc was observed binding in structure 4, it was never included in the crystallization trials (Table 2, structure 10). We reasoned that the D98N variant, for whatever reason, bound the ligand during its overexpression and purification from *Escherichia* *coli*. As a consequence, we crystallized the apoform of the D98N variant with the assumption that UDP-GlcNAc would be found in the allosteric pocket. The crystals were obtained at pH 6.0, and the model was refined to 1.8 Å resolution (overall *R*-factor of 18.1%). No ligands were observed. Note that the α-carbons for the apoform of the WT enzyme and the D98N variant correspond with an RMSD of 0.9 Å.

### Identification of exogenous ligands

**Figure 10:**
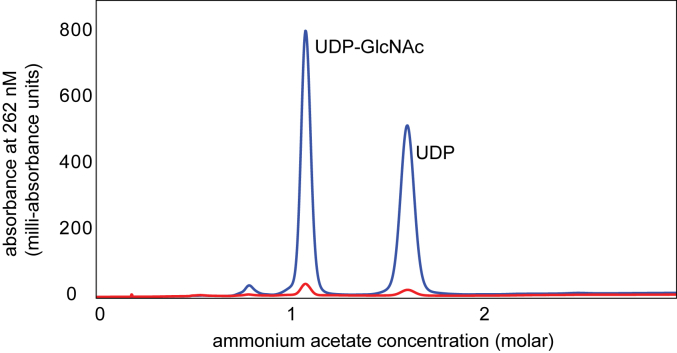
**Identification of the ligands bound to the D98N variant.** Upon denaturation and centrifugation of the D98N variant, the remaining solution was diluted and evaluated with an ÄKTA pure HPLC system using anion exchange. Shown is a plot of absorbance *versus* ammonium acetate concentration. The *blue* and *red lines* correspond to the results observed for the D98N variant and the WT protein, respectively.

Although the electron densities of the exogenous ligands in structure 4 were unambiguous, we utilized a combination of protein denaturation, HPLC, and mass spectrometry to verify their presence in the purified protein before the crystallization trials. As described in Experimental procedures, they were identified as UDP-GlcNAc and UDP. When the denaturation protocol was performed with the WT enzyme, the same two compounds were observed, but at only a couple of percent the abundance of that observed for the D98N variant as shown in Figure 10. On average, a protein solution of the D98N variant at a concentration of 0.12 mM contained 0.04 mM of UDP-GlcNAc and 0.03 mM of UDP that were released upon denaturation of the enzyme.

### Kinetic analysis

The kinetic parameters for the *T. thermophilus* 2-epimerase were evaluated by a discontinuous assay using an ÄKTA pure HPLC system. From the assays it was apparent that the addition of UDP-GlcNAc was required for the reactions to proceed. Evaluation of the data with GraphPad gave a *K*_*M*_ = 0.61 (±0.08) mM and *k*_cat_/*K*_*M*_ = 1100 (±200) M^−1^s^−1^. The calculated Hill coefficient was 2.5 (±0.6). By comparison, the reported catalytic efficiency for the enzyme from *E. coli* was 14,200 M^−1^s^−1^ (21). The order of magnitude less for the catalytic efficiency of the *T. thermophilus* 2-epimerase may possibly be due to the temperature (37 °C) utilized in our assays. The optimal growth temperature for *T. thermophilus* is ∼73 °C. The observed Hill coefficient for the *T. thermophilus* 2-epimerase is in keeping with that observed for the *E. coli* enzyme which was reported to be 1.8 (21).

## Discussion

The 2-epimerases that function on UDP-GlcNAc have caught the imagination of researchers for over 50 years. Indeed, in 1975, a report describing the enzyme from *E. coli* first appeared which was subsequently followed by a detailed biochemical analysis of the protein isolated from *Bacillus cereus* (24, 25). From these initial investigations, it was established that the optimum pH for catalytic activity for the enzyme was between 7.5 and 8.0, and that UDP-GlcNAc served as an activator. Indeed, a plot of the enzymatic rate as a function of substrate concentration appeared sigmoidal with a Hill coefficient of 1.8 (24, 25).

**Figure 11:**
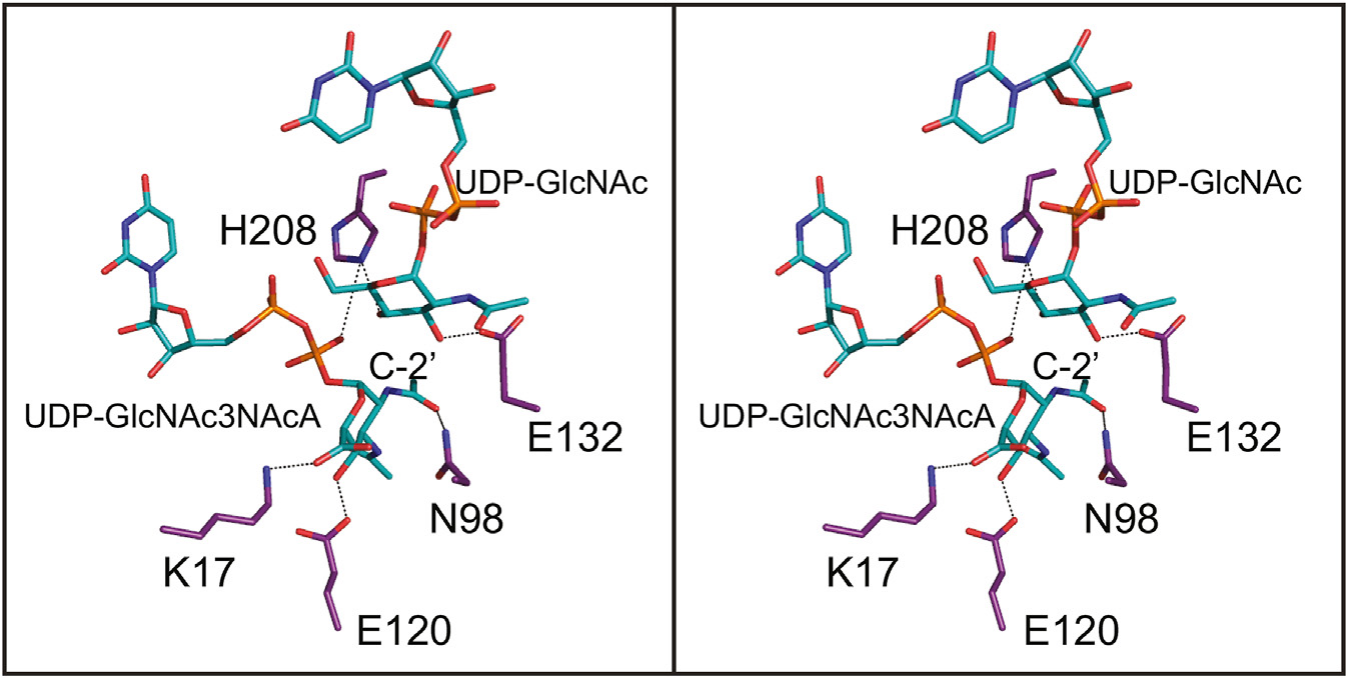
**Location of the amino acid residues targeted for biochemical analyses.** In the *E. coli* enzyme, Lys 15, Asp 95, Glu 117, Glu 131, and His 213 were examined for their role in catalysis. These correspond to Lys 17, Asn 98, Glu 120, Glu 132, and His 208 in the *T*. *thermophilus* enzyme as shown here.

The structure of the *E. coli* enzyme/UDP complex was determined at 2.5 Å resolution in 2000 (26). On the basis of this model, five highly conserved residues in the active site were targeted for investigation: Lys 15, Asp 95, Glu 117, Glu 131, and His 213 (21). These correspond to Lys 17, Asp 98, Glu 120, Glu 132, and His 208 in the *T. thermophilus* enzyme. The locations of these side chains are displayed in Figure 11. Given that our study reveals for the first time the manner in which the 2-epimerases simultaneously bind UDP-linked sugars in both the active site and the allosteric pocket, it is instructive to reconsider the roles of these residues. It was predicted that Lys 15 in the *E. coli* enzyme would be involved in substrate binding. In the *T. thermophilus* protein, Lys 17 is, indeed, within 3.2 Å of the C-6′ carboxylate. In the *E. coli* enzyme, mutation of His 213 to an asparagine residue resulted in a 30-fold increase in the value of *K*_M_,_app_ and a 50-fold decrease in the value of *k*_cat,app_, suggesting that the side chain plays some type of role in both binding and catalysis. As can be seen in Figure 11, His 208 in the *T. thermophilus* enzyme lies within hydrogen bonding distance to the C-4′ hydroxyl of the UDP-GlcNAc bound in the allosteric pocket. Its side chain is also located within 3.8 Å to a β-phosphoryl oxygen of the UDP-GlcNAc3NAcA ligand and thus it may function as a bridge between the two binding pockets. The activities of the D95N, E117Q, and E131Q variants in the *E. coli* 2-epimerase were so reduced that accurate kinetic parameters could not be determined. In the *T. thermophilus* enzyme, the carboxylate of Asp 98 (Asn 98 in structure 6) is positioned within 3.8 Å of the C-2′ carbon of the pyranosyl moiety where it most likely plays a critical role in proton abstraction. The side chain of Glu 120 forms an electrostatic interaction with the C-4′ hydroxyl of UDP-GlcNAc3NAcA. Given its location in the *T. thermophilus* enzyme, it is hard to envision it being involved in proton abstraction or donation. The position of Glu 132 is interesting in that it lies at 2.4 Å from the C-3′ hydroxyl of the UDP-GlcNAc ligand.

As shown in Figure 2, the proposed catalytic mechanism for the nonhydrolyzing 2-epimerases requires both a general base and an acid. From this investigation, as well as previous studies, it is clear that the conserved aspartate, Asp 98 in the *T. thermophilus* enzyme, is in the proper location to function as a catalytic base. The jury is still out regarding the identity of the catalytic acid. The Fisher laboratory has suggested that the conserved histidine, His 212, functions as the active site acid *via* a proton shuttle between it and the β-phosphate of the substrate (18). In the *T. thermophilus* enzyme, the side chain of His 208 is positioned at 2.7 Å from the C-4′ hydroxyl of UDP-GlcNAc and 3.7 Å of a β-phosphoryl oxygen of UDP-GlcNAc3NAcA as shown in Figure 11. In turn, this β-phosphoryl oxygen sits at 3.5 Å from the C-2′ carbon of UDP-GlcNAc3NAcA suggesting that a proton shuttle is feasible, at least based on the structural models presented here.

**Figure 12:**
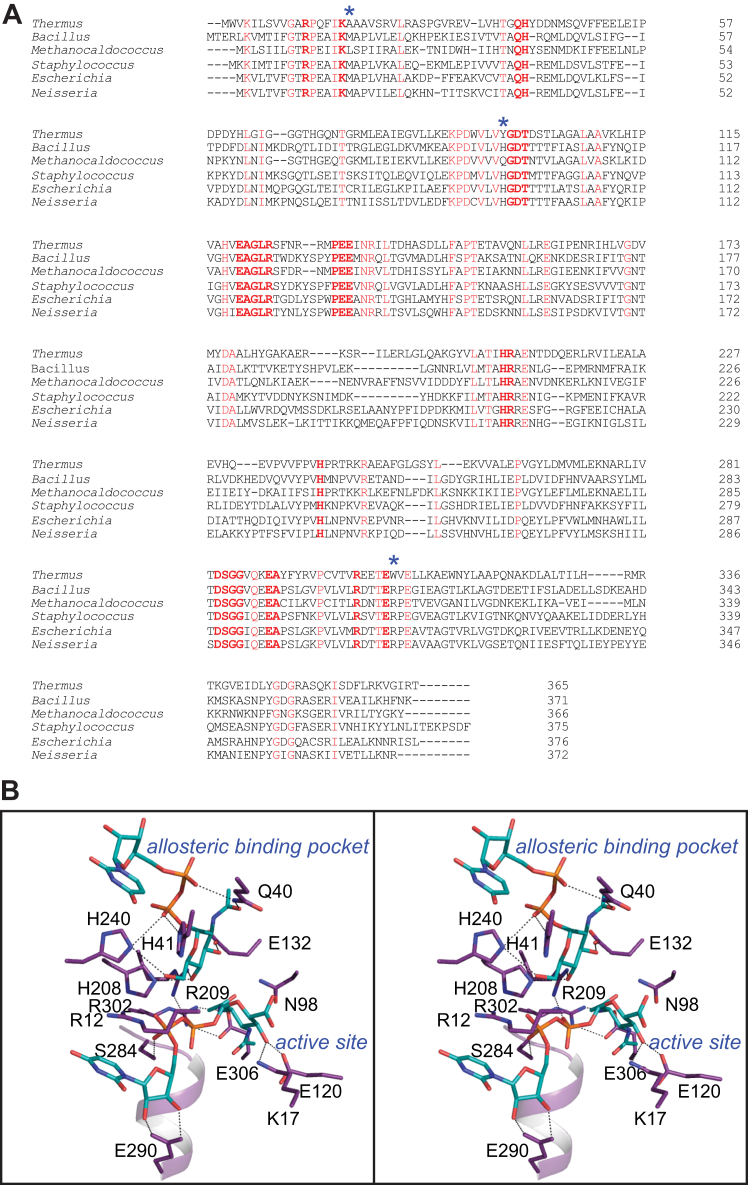
**Amino acid sequence alignment and location of conserved active site and allosteric binding residues in the *T***. ***thermophilus* 2-epimerase.** The amino acid sequence alignment, shown in (*A*), was performed with Clustal Omega. The shortened bacterial names in the alignment correspond to *T. thermophilus*, *B*. *anthracis*, *M*. *jannaschii*, *S*. *aureus*, *E*. *coli*, and *N*. *meningitidis*. Conserved residues are highlighted in *red*. The *blue asterisks* indicate the amino acid changes in the *T. thermophilus* enzyme that results because of its bulkier substrate. Of the 67 strictly conserved residues, 14 provide side chains that are involved in ligand binding and/or catalysis. The positions of these residues in the *T. thermophilus* 2-epimerase are shown in *stereo* in (*B*). Potential electrostatic interactions within 3.2 Å are indicated by the *dashed lines*.

The structures of the 2-epimerases from *E. coli*, *Bacillus anthracis*, *Methanocaldococcus jannaschii*, *Staphylococcus aureus*, and *Neisseria meningitidis* have been previously reported (16, 17, 18, 26, 27). Shown in Figure 12*A* is an amino acid sequence alignment of these enzymes against the *T. thermophilus* protein. There are 67 residues that are strictly conserved, many of which play structural roles. The locations of those involved in catalysis and/or UDP-sugar binding are shown in Figure 12*B*.

Those conserved residues in the catalytic site include Arg 12, Lys 17, Asp 98, Glu 120, Ser 284, Glu 290, Arg 302, and Glu 306. Specifically, the guanidinium group of Arg 12 forms a cation/π interaction with the uracil ring of the substrate. Lys 17 is involved in substrate binding by hydrogen bonding to the C-4′ hydroxyl of the substrate. Asp 98 is conserved because of its critical role as a catalytic base. As in the case of Lys 17, the side chain of Glu 120 forms an electrostatic interaction with the C-4′ hydroxyl of the substrate. The hydroxyl of Ser 284 hydrogen bonds with an α-phosphoryl oxygen of the substrate, and it is positioned at the positive end of a helix dipole moment that functions in neutralizing the charges on the pyrophosphoryl group. Glu 290 bridges the hydroxyl groups of the ribose of the substrate Finally, Arg 302 forms a salt bridge with Glu 306 thereby providing rigidity to one side of the active site.

Those conserved residues in the allosteric binding pocket include Gln 40, His 41, Glu 132, and His 240. The N^δ2^ of Gln 40 bridges the oxygen of the *N*-acetyl group and an α-phosphoryl oxygen of the allosteric ligand. Likewise, His 41 forms a hydrogen bond to a β-phosphoryl oxygen. Glu 132 hydrogen bonds to the C-3′ hydroxyl of the allosteric ligand and His 240 lies within hydrogen bonding distance to the C-6′ hydroxyl and a β-phosphoryl oxygen. Finally, His 208 and Arg 209 function as a bridge between the two UDP-sugar binding sites.

This is the first structural example of a 2-epimerase that functions on UDP-GlcNAc3NAcA rather than UDP-GlcNAc. These substrates differ by the substituent on the C-3′ carbon (an *N*-acetyl *versus* a hydroxyl group) and the hybridization about the C-6′ carbon (*sp*^2^ versus *sp*^3^). The question is then how does the *T. thermophilus* 2-epimerase accommodate these differences? The answer is rather simple. In all the enzymes that function on UDP-GlcNAc, there is a conserved arginine which hydrogen bonds to the C-3′ hydroxyl group. This is replaced in the *T. thermophilus* enzyme by Trp 307 (Fig. 12*A*). In the enzymes that function on UDP-GlcNAc, there is typically a leucine or methionine residue which in the *T. thermophilus* protein is Ala 18. Likewise, in the former enzymes there is either a histidine or a glutamine which in the *T. thermophilus* enzyme is Tyr 96. Whereas in the typical 2-epimerases the histidine or glutamine residues project toward the C-6′ hydroxyl, in the enzyme studied here the side chain of Tyr 96 flips out of the active site. This movement would require a smaller residue, hence the change from a methionine or leucine to an alanine.

In conclusion, high resolution structures of a 2-epimerase that functions on UDP-GlcNAc3NAcA have now been determined. By the judicious use of site-directed mutagenesis, we have defined the active site architecture before and after catalysis. In addition, we have demonstrated that this enzyme is, indeed, allosterically regulated by UDP-GlcNAc. Importantly, to the best of our knowledge, this is the first time in this family of enzymes that UDP-linked sugars have been observed simultaneously binding in both the active and the allosteric sites, which provides novel insight into these fascinating proteins.

## Experimental procedures

### Protein expression and purification

The gene encoding the *T. thermophilus* 2-epimerase (WP_011172736, locus tag TT_RS01455) was cloned from genomic DNA (American Type Culture Collection BAA-163D) using PrimeSTAR HS DNA Polymerase (Takara BIO, Clontech Laboratories). It was subsequently inserted into the pET31 vector to give a construct with a C-terminal polyhistidine tag (LEHHHHHH), and into the pET28jt vector (28) to produce a construct with a recombinant tobacco etch virus (rTEV)-cleavable N-terminal polyhistidine tag (MGSSHHHHHHSSENLYFQGH). These plasmids were utilized to transform Rosetta2(DE3) *E. coli* cells for protein expression. Cultures in lysogeny broth were grown at 37 °C with appropriate antibiotics until absorbances of 0.8 were obtained as measured at 600 nm. The cultures were cooled, isopropyl β-d-1-thiogalactopyranoside was added to a final concentration of 1 mM, and the cultures were then allowed to express protein at room temperature (21 °C) for 24 h after induction.

The cells were harvested by centrifugation and frozen as pellets in liquid nitrogen. These pellets were subsequently disrupted by sonication on ice in a lysis buffer composed of 50 mM sodium phosphate, 20 mM imidazole, 10% (w/v) glycerol, and 300 mM NaCl (pH 8.0).

The lysates were cleared by centrifugation, and all proteins were purified at 4 °C utilizing Prometheus Ni-NTA agarose (Prometheus Protein Biology Products) according to the manufacturer’s instructions. All buffers were adjusted to pH 8.0 and contained 50 mM sodium phosphate, 300 mM NaCl, and imidazole concentrations of 20 mM for the wash buffer and 300 mM for the elution buffer. One half of the protein purified with the N-terminal polyhistidine was digested with rTEV protease for 48 h at 4 °C to remove the tag. The rTEV protease and remaining tagged proteins were removed by passage over Ni-NTA agarose, and the tag-free protein was dialyzed against 10 mM Tris (pH 8.0) and 200 mM NaCl. Purified tagged protein pools were similarly dialyzed. The proteins were concentrated to 11 to 12 mg/ml based on an extinction coefficient of 1.02 (mg/ml)^−1^ cm^−1^.

The site directed variant, D98N was made *via* the QuikChange method of Stratagene. It was expressed, purified, and concentrated in the same manner as the WT enzyme.

### Ligand synthesis

UDP-GlcNAc and UDP-GlcNAc3NA (Figure 1) were prepared as previously described (29, 30). UDP-GlcNAc3NAcA was synthesized according to Hofmeister *et al.*, (30). The reaction for synthesizing UDP-GlcNAc3NAcA was scaled up by starting with 0.5 g of UDP-GlcNAc3NA to ensure that all experiments used the same ligand preparation. The acetyl coenzyme A required for this was synthesized as previously described (29).

### Analysis of the ligand binding to the D98N variant

It became apparent from the electron density maps of the D98N variant that exogenous ligands were being copurified. To determine the identity of the ligands, 10 mg of the D98N variant was denatured by heating and subsequent removal of the precipitate. The remaining solution was diluted and evaluated with an ÄKTA pure HPLC system at 262 nm with a 1 ml Resource Q column using a gradient of 0 to 3 M ammonium acetate at pH 4.0 to separate small molecules released upon protein denaturation. Two major peaks were observed. Along with known compounds tested using the same HPLC protocol, these peaks were shown to correspond to UDP-GlcNAc (elution at 1.1 M ammonium acetate) and UDP (elution at 1.7 M ammonium acetate). When the denaturation was performed with the WT enzyme, the same two compounds were observed but at only a couple of percent the abundance of that observed for the D98N variant. The identity of the compounds in the two peaks were further confirmed by electrospray ionization mass spectrometry (parent ion at *m/z* 606.1 for UDP-GlcNAc and *m/z* 403.0 for UDP).

For the determination of amount of bound ligand, 1 ml of the D98N variant at 5 mg/ml (0.12 mM) was heat denatured for 10 min to release the bound molecules. The solution was then centrifuged for 5 min at 20,000*g* to remove the precipitated protein and subsequently filtered to ensure all particulates were removed. Hundred microliters of the filtered solution was diluted with 2.5 ml of water and 2 ml loaded onto a 1 ml Resource-Q column and separated as described above. All three variations of the enzyme (N terminally tagged, C terminally tagged, and tag-free) were tested. Peak areas were correlated to concentration after generation of a calibration curve relating concentration to chromatogram peak area using solutions of known concentration treated in the same manner as the samples generated from the protein solutions. All protein constructs showed that approximately the same amount of UDP and UDP-GlcNAc were bound. On average a protein solution of the D98N variant at a concentration of 0.12 mM contained 0.04 mM of UDP-GlcNAc and 0.03 mM of UDP that were released upon denaturation of the enzyme.

### Crystallization, X-ray data collection, and structural analyses

Crystallization conditions were determined by the hanging drop method of vapor diffusion using a 144 condition laboratory-based sparse matrix screen. The WT enzyme in the presence of 5 mM UDP-GlcNAc3NAcA was surveyed first. Crystals were obtained between pH 5.0 to 9.0 under a variety of conditions at room temperature. The first X-dataset was collected from crystals grown at pH 9.0 from 18 to 22 % (w/v) poly(ethylene glycol) 3350. Crystals were prepared for X-ray data collection by transferring them to a cryoprotectant solution composed of 30% (w/v) poly(ethylene glycol) 3350, 200 mM NaCl, 5 mM UDP-GlcNAc3NAcA, 15% (v/v) ethylene glycol, and 100 mM CHES (pH 9.0). They belonged to the monoclinic space group *P2*_1_ with unit cell dimensions of *a* = 53.6 Å, *b* = 128.6 Å, *c* = 58.9 Å, and β = 111.2^o^. The asymmetric unit contained one dimer. X-ray data were collected using a BRUKER D8-VENTURE sealed tube system equipped with HELIOS optics and a PHOTON II detector. The X-ray datasets were processed with SAINT and scaled with SADABS (Bruker AXS). This structure was solved by molecular replacement using Protein Data Bank (PDB) entry 3BEO as a search model (16). Iterative cycles of model-building with Coot (31, 32) and refinement with REFMAC (33) led to a final X-ray model with an overall *R*-factor of 18.3%. Relevant X-ray data collection statistics are listed in Table 2.

Crystallization conditions, X-ray data collection statistics, and refinement statistics for all other complexes are provided in Table 2. Note that the crystallization conditions for all of the complexes were initially examined under the same 144 sparse matrix screen. Cryoprotection for the various crystals involved increasing the amount of precipitant used to 10% w/v higher that the crystallization condition (*i.e.*, if the crystals were grown from 15% (w/v) poly(ethylene) glycol, the percentage poly(ethylene) glycol in the cryoprotectant solution was 25% (w/v)) with the addition of 15% (v/v) ethylene glycol). Structures were determined *via* Fourier difference techniques or molecular replacement with the software packager PHASER (34).

### Kinetic analysis

Kinetic parameters were evaluated *via* a discontinuous assay using an ÄKTA pure HPLC system equipped with a 1 ml Resource-Q column. The 1 ml reactions contained 50 mM HEPPS (pH 8.0), 0.35 mM UDP-GlcNAc, and UDP-GlcNAc3NAcA ranging from 0.05 to 7 mM. The presence of UDP-GlcNAc was required for the reactions to proceed. Tests varying UDP-GlcNAc showed that when using 44 μM of the *T. thermophilus* 2-epimerase, the reaction rates remained constant as long as the UDP-GlcNAc concentration was at least 100 μM.

These above described solutions were incubated at 37 °C for 10 min, and the reactions were initiated with the addition of the tag-free 2-epimerase to a final concentration of 44 μM. Reactions were maintained at 37 °C and 150 μl samples were taken over a period of 40 to 120 min. Samples were immediately quenched by the addition of 6 μl of 6 M HCl followed by the addition of 200 μl chloroform with vigorous mixing. The samples were then centrifuged for 2 min at 20,000*g*, and 120 μl of the aqueous phase was removed for HPLC evaluation. After the addition of 2.3 ml of water, 2 ml of the diluted samples were loaded onto the Resource-Q anion exchange column. The column was then washed with 3 ml 0.9 M ammonium acetate (pH 4.0), and the reaction products were separated using a 15 ml gradient of 0.9 to 1.8 M ammonium acetate (pH 4.0). Reaction rates were determined by calculating the amount of product formed based on the HPLC trace peak area. The area was correlated to concentration *via* a calibration curve created with standard samples that had been treated in the same manner as the reaction aliquots. Evaluation with GraphPad gave *K*_*M*_ = 0.61 (±0.08) mM and *k*_cat_/*K*_M_ = 1100 (±200) M^−1^s^−1^. The calculated Hill coefficient was 2.5 (±0.6).

## Data availability

X-ray coordinates and structure factors have been deposited in the Protein Data Bank under accession codes: 8SXV, 8SXY, 8SY0, 8SY9, 8SYA, 8SYB, 8SYD, 8SYE, 8SYH, 8SXW.

## Conflict of interest

The authors declare that they have no conflicts of interest with the contents of this article.
